# Modulation of manual preference induced by lateralized practice diffuses over distinct motor tasks: age-related effects

**DOI:** 10.3389/fpsyg.2014.01406

**Published:** 2014-12-04

**Authors:** Rosana M. Souza, Daniel B. Coelho, Luis A. Teixeira

**Affiliations:** ^1^Department of Physical Therapy, Federal University of São CarlosSão Carlos, Brazil; ^2^Human Motor Systems Laboratory, Biodynamics of Human Movement, University of São PauloSão Paulo, Brazil

**Keywords:** handedness, non-preferred hand use, right-handers, diffusion of manual preference, confidence

## Abstract

In this study we investigated the effect of use of the non-preferred left hand to practice different motor tasks on manual preference in children and adults. Manual preference was evaluated before, immediately after and 20 days following practice. Evaluation was made with tasks of distinct levels of complexity requiring reaching and manipulation of cards at different eccentricities in the workspace. Results showed that left hand use in adults induced increased preference of that hand at the central position when performing the simple task, while left hand use by the children induced increased preference of the left hand at the rightmost positions in the performance of the complex task. These effects were retained over the rest period following practice. Kinematic analysis showed that left hand use during practice did not lead to modification of intermanual performance asymmetry. These results indicate that modulation of manual preference was a consequence of higher frequency of use of the left hand during practice rather than of change in motor performance. Findings presented here support the conceptualization that confidence on successful performance when using a particular limb generates a bias in hand selection, which diffuses over distinct motor tasks.

## Introduction

Human laterality has been understood traditionally from the perspective that there is a dominant hemisphere for motor control, leading to intermanual performance asymmetry favoring the dominant hand (Annett, 1972; Levy and Nagylaki, 1972; McManus, 1985). Based on intermanual performance asymmetry, a lateral bias of use is established with the dominant hand becoming the preferred one to perform motor actions in general. From this perspective, manual preference to perform voluntary movements is expected to be a stable characteristic of motor behavior. However, contradictory to the expectation of stability of manual preference, different investigations have shown that the relative frequency of use of the right/left hand is malleable, and that it depends on lateralized experiences. Malleability and generalization of manual preference as a result of lateralized motor experiences have been investigated through experimental approaches in children and adults. Teixeira and Teixeira (2007) provided right-handed adults with practice for the non-preferred left hand in sequential touches between the fingers and the thumb, assessing variation of manual preference afterwards. Evaluation of manual preference revealed that 7 out of 10 participants shifted from right to left hand preference to perform the specific experimental task immediately after practice. More specifically, at that moment four participants declared that, if they had opportunity to choose, they would select exclusively their left hand to perform the experimental task, and three other participants would use their left hand in most trials. Thus, practice with the left hand in the experimental task created a specific manual preference incongruent with the global preference for the right hand to perform daily living motor tasks. That effect was retained over 1 month of rest, showing to be a persistent one. An additional point of interest in those results was that no correlation was found between manual preference and intermanual performance asymmetry. In fact, there were some cases of contradictory relationship between manual preference and performance asymmetry. In a follow-up experiment, Teixeira and Okazaki (2007) evaluated the extent to which lateralized practice induces modulation of manual preference not only for the specifically practiced task but also for distinct motor tasks. To evaluate generalizability of modulation of manual preference by lateralized practice, we provided adult right-handers with practice of a single sequence of fingers movements using their non-preferred left hand, and assessed its effect on manual preference for two other sequences of fingers movements. Results revealed that repeated use of the non-preferred left hand during practice led to modulation of manual preference both for the specific task and for another one having the same sequential structure as that practiced. Similarly to what was observed for the practiced task, the effect of generalization was persistent over time. Of particular interest, analysis of movement time revealed that practice with the non-preferred hand led to similar performance gains between the hands. Therefore, shift of manual preference was not associated with intermanual performance asymmetry. Results from these two experiments revealed the malleability of manual preference even in right-handed adults, an age group having stable manual preference in daily living tasks, and that modulation of manual preference induced by unimanual practice of one task can affect manual preference in distinct motor tasks.

To assess the extent to which use of a single hand in daily living activities is able to bias manual preference in non-practiced tasks, we (Teixeira et al., 2010) provided 3- and 4-year-old children with motor experiences with their non-preferred left hand in different tasks requiring pencil manipulation. Manual preference was probed by evaluating the hand chosen to perform a simple task of reaching and grasping a pencil, and a complex task requiring reaching, grasping and inserting the pencil into a small orifice. Grasping targets were positioned at the midline and at different eccentricities in both sides of the child's workspace. As expected, before practice the children showed a noticeable preference for using their right hand, particularly when reaching for targets positioned at the midline and at different points of the right hemispace. Following practice, the children manifested higher rates of use of the left hand at the midline and at right-sided target positions. Modulation of manual preference was expressed through prevalent use of the left hand across target positions, or lower frequency of use of the right hand in comparison with the pretest. That effect was observed for both the simple and complex motor tasks. From these findings, we forged the concept of “diffusion of manual preference” to convey the notion that a lateral bias developed by predominant use of a single hand to perform one or a group of motor actions spreads over other tasks having similar movement control requirements.

This series of experiments oriented to understand the effect of systematic use of a single hand on manual preference suggest that shift of manual preference following practice is not necessarily associated with an improved status of the non-preferred hand in intermanual performance asymmetry. It might be thought from the findings of modulation of manual preference by use that hand selection is biased by the confidence one acquires that successful performance can be achieved with a particular hand based on its history of use. Support for this conceptualization has been provided by Stoloff et al. (2011) through manipulation of the perceived rate of success in the performance of an aiming task with the preferred or non-preferred hands. In this experiment, they increased the probability that trials performed with the non-preferred hand received feedback indicating successful performance, and did the opposite to performance with the preferred hand by increasing the frequency of sense of failure when using that hand. This procedure induced a higher proportion of use of the non-preferred left hand, an effect that persisted following the end of feedback manipulation. Participants reported to be concerned regarding performance with their non-preferred hand at the experiment onset, whereas they declared to have become confident on using that hand over trials. Additionally, most participants declared that the task seemed to have become easier to be performed with their left hand across trials. These results support the conjecture that confidence achieved by hands use modulates manual preference to perform motor actions.

In the present experiment, we evaluated the extent to which higher frequency of use of the non-preferred hand in several motor tasks modulates manual preference in different probing tasks. For this evaluation, in addition to adults, we assessed 8- to 10-years-old children because this age has been shown to be associated with the most consistent use of the preferred right hand (Bryden and Roy, 2006; Doyen et al., 2008; Hill and Khanem, 2009). By selecting these age groups, we aimed at making a strict test of the effect of lateralized practice on manual preference by using participants who can be considered to be the most difficult ones to induce increased use of the non-preferred hand. Manual preference was probed by using targets arranged at different points in the left and right sides in egocentric coordinates of the workspace (cf. Bishop et al., 1996). This setup has proved to provide a discriminant assessment of consistency of manual preference, since right-handers have been observed to use their preferred hand consistently to reach for targets placed either at the midline position or in the right hemispace, whereas targets positioned in the left hemispace induce increased use of the left hand as eccentricity of target position is increased. This effect has been observed in children (Gabbard et al., 2001; Leconte and Fagard, 2004, 2006; Bryden and Roy, 2006; Carlier et al., 2006; Doyen et al., 2008; Hill and Khanem, 2009), as well as in adults (Bishop et al., 1996; Bryden et al., 2000; Stins et al., 2001; Bryden and Roy, 2006). To increase the discrimination power of the evaluation of manual preference, we also compared tasks of different complexities. Complex tasks have been shown to lead to higher frequency of use of the preferred hand both in children (Rostoft et al., 2002; Fagard and Lockman, 2005; Mayer and Bryden, 2008; Hill and Khanem, 2009) and in adults (Calvert and Bishop, 1998; Mamolo et al., 2004). Thus, it is expected that manual preference in complex tasks is less amenable to modulation by hands use than in simple tasks. An additional point of original interest in the present investigation was evaluation of manual preference in parallel with analysis of movement kinematics, in order to acquire further insight into the role of intermanual performance asymmetry in hand selection. Considering that the practice tasks were different from those evaluated and that there was no emphasis on performance improvement during practice, we expected to find no modification of intermanual performance asymmetry resulting from practice. Based on the concept of diffusion of manual preference, we hypothesized that practice of different motor tasks using the non-preferred hand induces increased preference of that hand to perform distinct motor tasks.

## Materials and methods

### Participants

Eighteen children (*n* = 9 for each gender), age range 8–10 years old (*M* = 9.2 years, *SD* = 0.6), and 18 adults (females *n* = 11, males *n* = 7), age range 18–28 years old (*M* = 22.5 years, *SD* = 3.1), volunteered for this study. Participants self-declared to be right-handed for handwriting and for daily living manual tasks. In addition, children had right-handedness confirmed by the respective teacher or parent. Adults and children's parents signed an informed consent form, as approved by the local university ethics committee.

### Task and equipment

**Figure 1:**
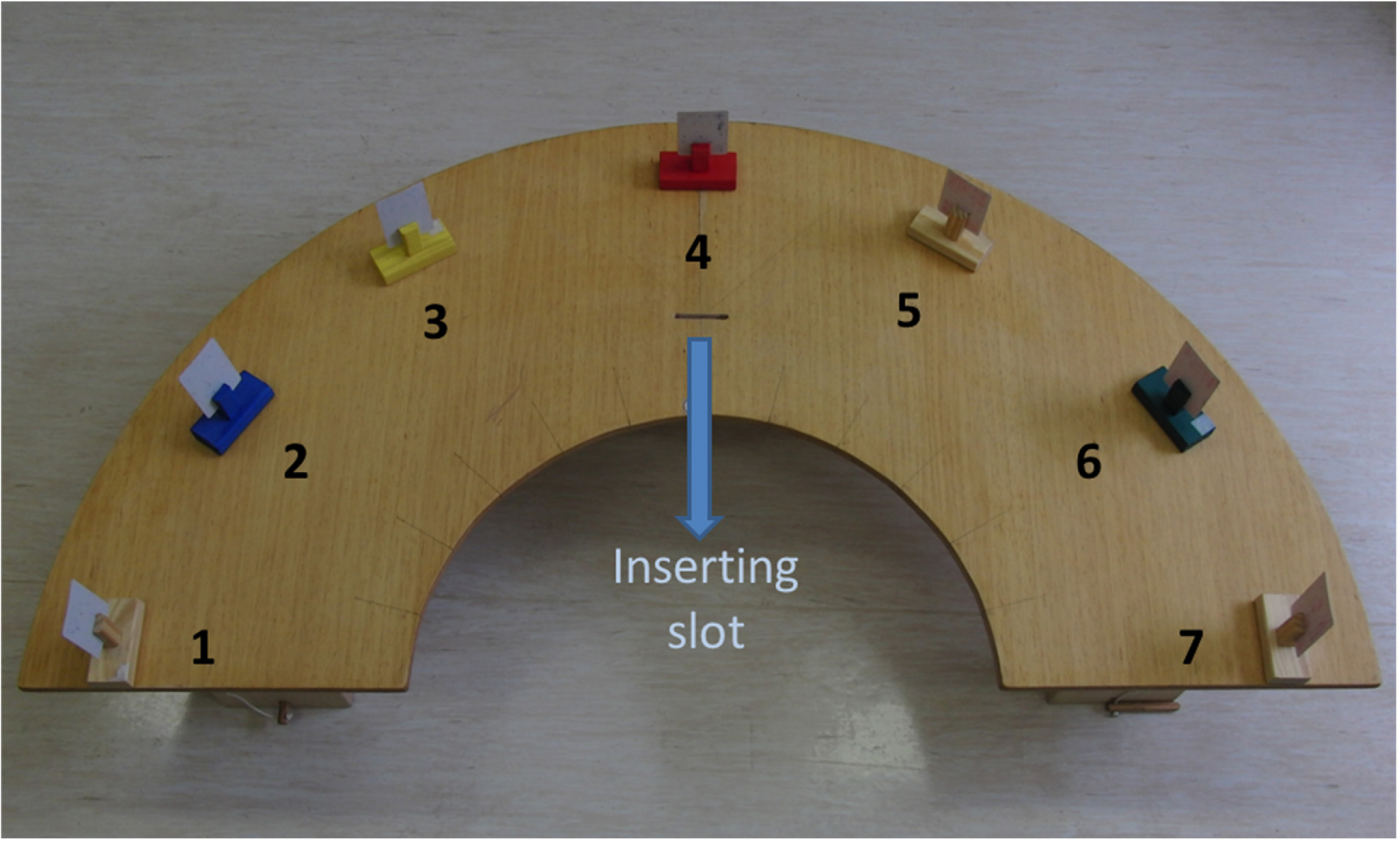
**Over-head perspective of the table surface showing the spatial arrangement of the cards (supported by holders) ranging from the leftmost (number 1) to the rightmost (number 7) position**. Aligned with the position number 4 is indicated the slot used for inserting the cards in the complex task. Participants sat at the round opening of the table.

For probing manual preference we used two tasks differing in complexity ^1^. The simple task consisted of reaching, grasping and laying down cards arranged at different eccentricities in the workspace on a supporting half-moon shaped table. Paper cards (8.5 × 5 cm) were supported in vertical orientation by cardholders at seven positions regarding participants' egocentric coordinates. Card positions were midline, three positions in the left side, and three positions in the right side. Cards were placed on an imaginary semicircle, 25 cm far from the proximal border of the table, with 30° spacing between adjacent cards (approximately 25 cm of linear distance). Those positions were numbered from 1 to 7, leftmost and rightmost respectively, with the number 4 corresponding to the midline position (Figure 1). In the complex task, participants were to grasp the card, transport and insert it into a slot. The slot was 6-cm long, 3-mm wide, being oriented parallel to participants' frontal plane. It was located 12 cm away from the table's proximal border, at the midline position. Initial position for the hands was on the participant's lap, supporting each hand on the ipsilateral leg. Adults were sat at a regular chair keeping their hip and knees flexed at 90° approximately, while children were sat at a height adjustable chair, keeping the same position as described for the adults.

For motor performance assessment, we used a modified version of the complex task. For this task, the card was placed at the central position, 20 cm far from the slot. Initial position was supporting the active hand on the table, 30 cm far from the card, near the proximal border of the table, aligned with the central position. The index finger and thumb were kept touching each other, with the hand oriented in a comfortable neutral position. Participants were to use their index finger and thumb in a pinch-like movement to pick up the card, making contact with the card at its upper border. For kinematic analysis, reflective markers were attached to the participants' index finger and thumb nails, and to the center of the radiocarpal joint of both hands. Four optoelectronic cameras (MX3+, Vicon) were used for acquisition of kinematic data.

### Experimental design and procedures

The experiment was conducted in four phases: pretest, practice, posttest, and retention. In the pretest, we assessed manual preference and performance asymmetry for the simple and complex tasks. Evaluation of manual preference was made through sets of 7 trials, one trial for each card position within a set. Manual preference for each task complexity was assessed through four sets of trials, corresponding to four trials for each card position, as it has become standard from previous investigation (Souza et al., 2012; Pogetti et al., 2014). In total, participants performed 28 trials for each task complexity. Sequence of card positions was pseudorandomized in each set of trials, with the card to be grasped being indicated by the experimenter through verbal instruction. Movements were self-paced following a command to initiate a trial. Participants were informed that they could freely select the right or the left hand to perform the tasks. Intertrial intervals within a set of trials were approximately 10-s long, and intervals between sets of trials were approximately 30-s long. For evaluation of motor performance, participants performed four trials of the modified complex task with each hand. Sequence of task complexities and hands were counterbalanced within each group (see groups description in the following).

**Figure 2:**
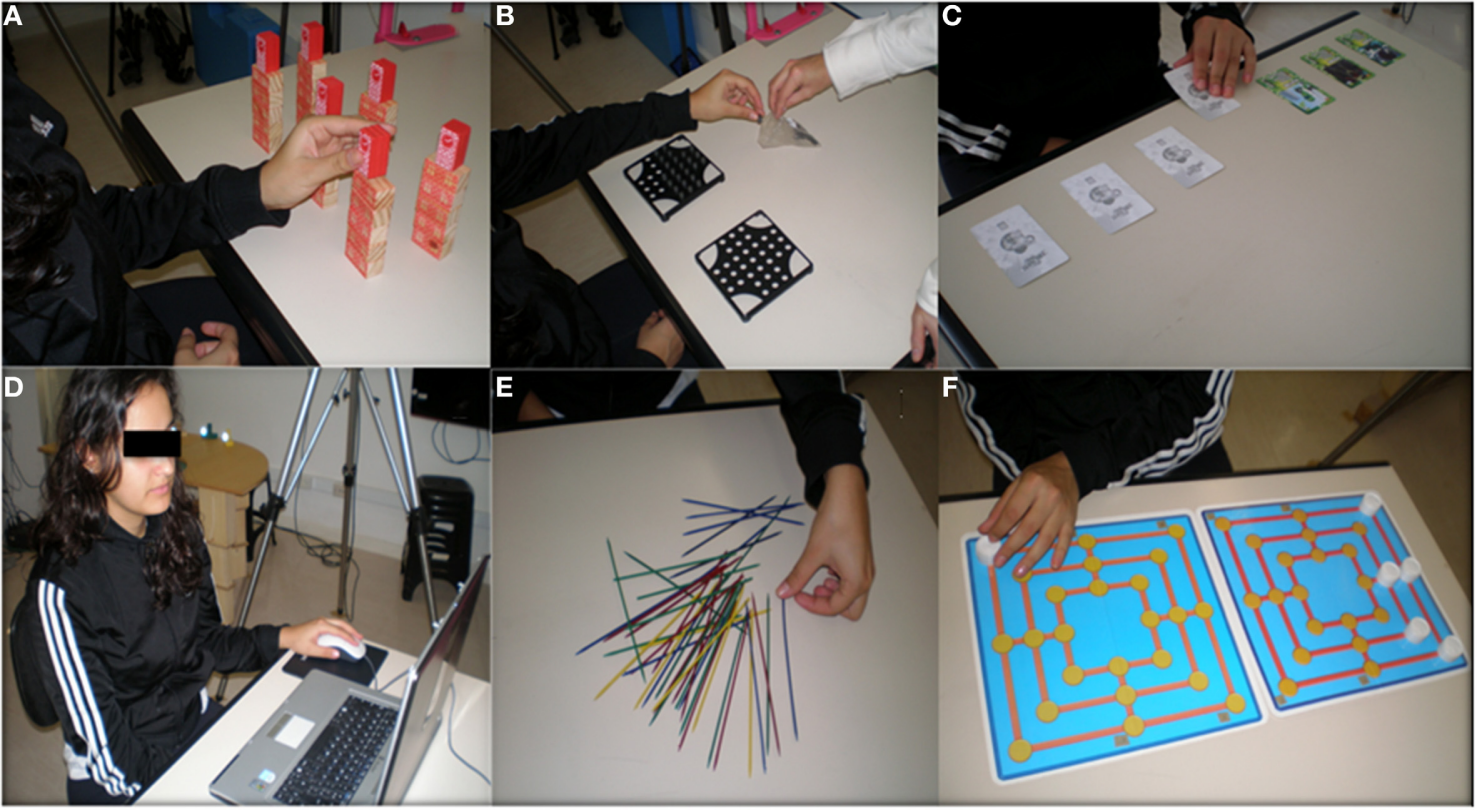
**Tasks used during practice. (A)** stacking wooden blocks, **(B)** grasping and inserting small balls into openings on a board, **(C)** sequential upside down turning of cards on a table, **(D)** tracking a small moving target on the computer screen using a computer mouse, **(E)** sequential picking up of sticks, and **(F)** moving bi-directionally 2-cm diameter round plastic pieces between left-right and proximal-distal positions aiming at spatial targets on a board.

In the practice ^2^ phase, half the participants of each age group were assigned to an experimental or control group, with similar numbers of males and females in each group. During this phase, experimental groups practiced several reaching and manipulative tasks using their non-preferred left hand. Practice tasks consisted of (A) grasping wooden blocks scattered on a table and stacking them into small buildings; (B) employing index finger and thumb pinch-like movements, grasping small (1-cm diameter) balls in a container, transporting and inserting them into round openings on a board; (C) sequential sliding and turning upside down cards on a table; (D) tracking a small moving target on a computer screen through manipulation of a computer mouse; (E) sequential picking up of sticks scattered on a table, with the restriction of not moving the other sticks; and (F) moving bidirectionally 2-cm diameter round plastic pieces between left-right and proximal-distal positions aiming at spatial targets on a board (Figure 2). Practice tasks, thus, had some motor control requirements similar to those of the probing tasks, involving reaching and manipulation, but they were distinct in terms of movement specificity. Although the practice tasks required grasping, transporting and inserting skills, features like manipulated objects, range of motion across the workspace and movement amplitude were different between the practice and probing tasks. Those tasks were practiced with the participant sat at a table supporting the described task-related material. While the left hand was active in performing one task, the right hand was maintained motionless supported on the participant's lap. Participants practiced during 20 min per day twice a week, during 3 weeks, totalizing 120 min of practice in six sessions. In each session, four of the described tasks were practiced during 5 min each one. The six motor tasks were varied in a balanced way between sessions of practice, accumulating 20 min of practice for each one of the tasks at the end of this phase. Participants were instructed to perform the tasks at a self-paced rhythm, without emphasis on movement improvement across trials either in terms of accuracy or time. They were not provided with augmented feedback. Rest intervals of 30 s were introduced in the transition between sets of trials for each task. Experimental groups of both ages practiced the same tasks under experimenter's supervision. During the practice phase control groups had no activities associated with the experiment. Posttest was made 5 min after a passive rest interval following the last practice session, and retention was tested 20 days following posttest. In posttest and retention, procedures were the same as described for the pretest. Cameras acquisition frequency for recording of kinematic data was set at 240 Hz.

### Data analysis

Manual preference was measured through the following equation: (R − L)/(R + L), in which *R* represents number of trials performed with the right hand, and *L* represents number of trials performed with the left hand. This equation was applied separately for each card position by task, individually for each participant. The score varied between −1 and 1, in which negative values indicate prevalent use of the left hand, and positive values prevalent use of the right hand.

To assess motor performance, data were first digitalized through the Vicon Nexus software, and then data were analyzed through custom-made Matlab® (Mathworks, Inc., Natick, MA) routines. Prior to acquisition of kinematic data, participants performed three static trials keeping a block of six cards between the index finger and thumb. The average distance between finger markers was considered as the criterion to determine the moment at which the card was grasped at the end of reaching, and the time of fingers aperture to insert the card into the slot in the complex task. Movement analysis was divided into two components: reaching and transporting. Reaching initiation was defined as the moment that wrist velocity reached 5% of peak velocity, and its end was defined as the moment that between-finger distance achieved the criterion value. Initiation of the transporting component was defined as the moment of card grasping and its end at the time that the thumb was inside a virtual area delimited by a radius of 140 mm on the horizontal plane with its center at the middle of the slot, and the distance between the thumb's marker and a marker bordering the slot was equal to 70 mm in the *Z* coordinate. The following kinematic variables were evaluated for the reaching and transporting movement components: movement time; straightness score, given by the ratio of the distance between the initial wrist position and the card by hand displacement; number of movement units, given by the frequency of peaks sided by valleys in the velocity curve for which differences in instantaneous velocities were greater than 1 cm/s. Raw data was filtered through a dual-pass fourth order Butterworth filter with cutoff frequency set at 10 Hz.

## Results

### Manual preference

**Figure 3:**
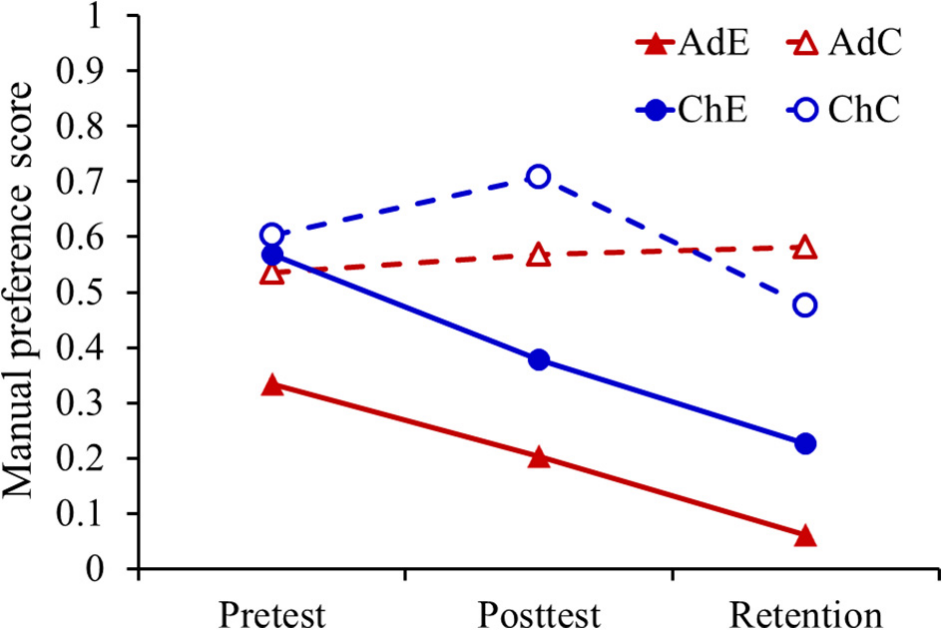
**Comparison of experimental and control age groups (Ad, adults; Ch, children; E, experimental; C, control), based on the global score of manual preference computed across all card positions, showing a trend of the experimental groups toward higher use of the left hand in posttest and retention as compared with pretest**.

In order to have a perspective of the general effect of practice using the non-preferred left hand, in a preliminary analysis we pooled individual data of all target positions and task complexities to compare scores of manual preference across tests. Figure 3 shows that descriptive analysis, suggesting a global trend toward reduced preference of the right hand in posttest and retention for both the adults and children experimental groups, whereas the respective controls showed a stable manual preference of their right hand. Decreased scores of manual preference following lateralized practice were due to the fact that at individual level most participants showed modulation of their hand preference. Analyzing enduring results of retention in comparison with the pretest, we observed the following: four participants maintained predominant use of their right hand but with decreased frequency, two shifted manual preference as indicated by predominant use of the left hand across target positions, whereas three participants showed no sensibility to left hand use during practice, maintaining the same manual preference between evaluations. These numbers were the same for each age group. For the statistical analysis presented in the following, we will describe significant effects only.

**Figure 4:**
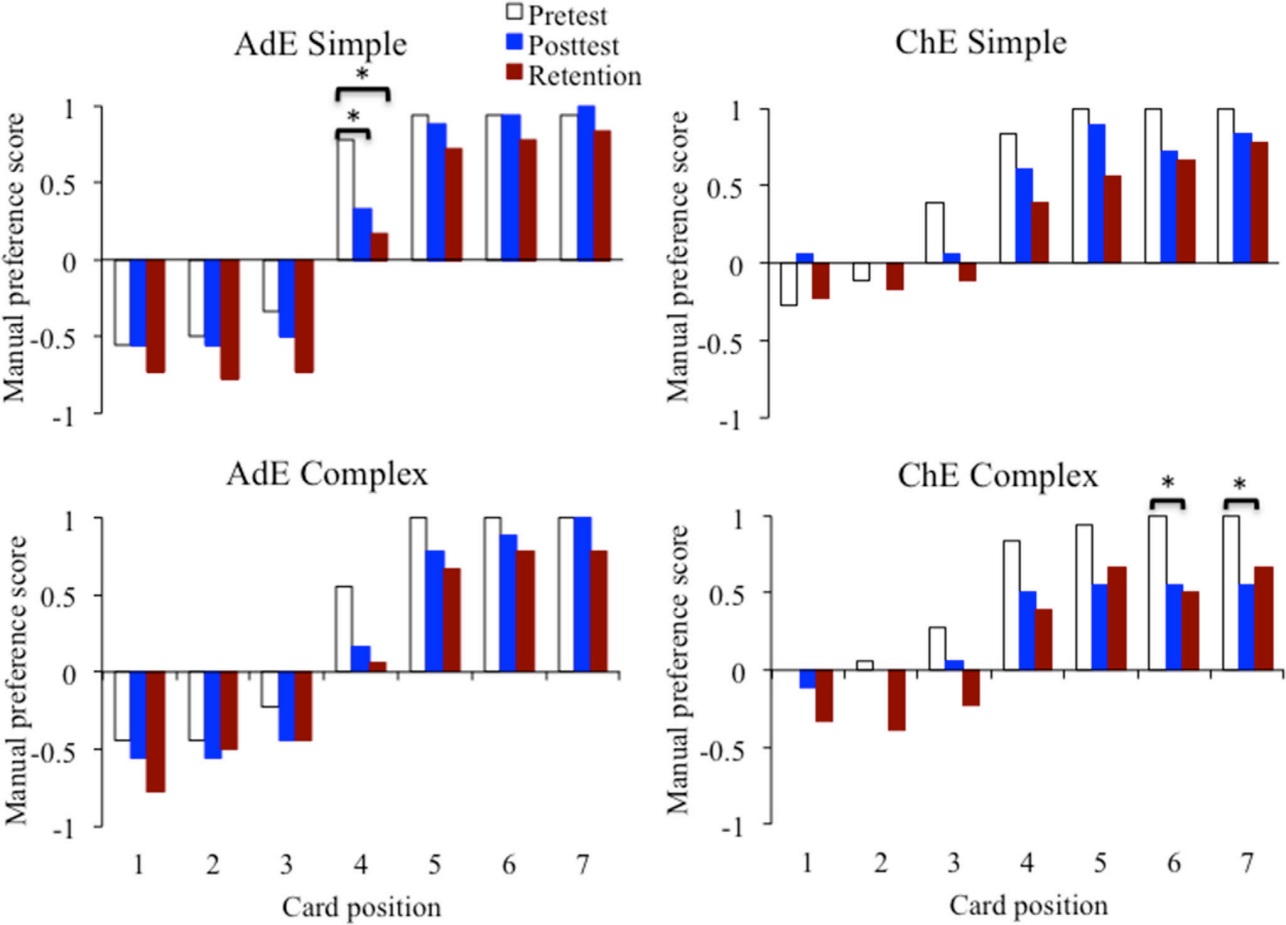
**Scores of manual preference for each card position, comparing values across tests (pretest, posttest, and retention)**. Left side panels show results for the adults and right side panels results for the children, upper panels show results for the simple and lower panels for the complex task. Statistically significant results are indicated by means of asterisks.

Statistical analysis of variation of scores of manual preference across tests was made separately for each card position by task complexity through Wilcoxon paired comparisons. Descriptive data from this analysis is shown in Figure 4. An overview of the several comparisons of task complexity by target position in that figure suggests that manual preference was affected by increased left hand use in most conditions for both the adults and children experimental groups. However, results showed significant effects of test for specific positions only, which were distinct between the experimental age groups. For the adults, significant differences were found on the simple task at the midline position: pretest × posttest (*Z* = 1.99, *p* = 0.05), and pretest × retention (*Z* = 2.02, *p* = 0.04). For the children, significant differences were found in the complex task: positions 6 and 7, pretest × posttest (*Z* = 2.07, *p* = 0.04, for both comparisons). Even though differences between pretest and retention did not reach statistical significance both for positions 6 (*p* = 0.07) and 7 (*p* = 0.11), no significant differences were found between posttest and retention comparisons (*p*-values > 0.58) for these positions.

### Movement kinematics

**Figure 5:**
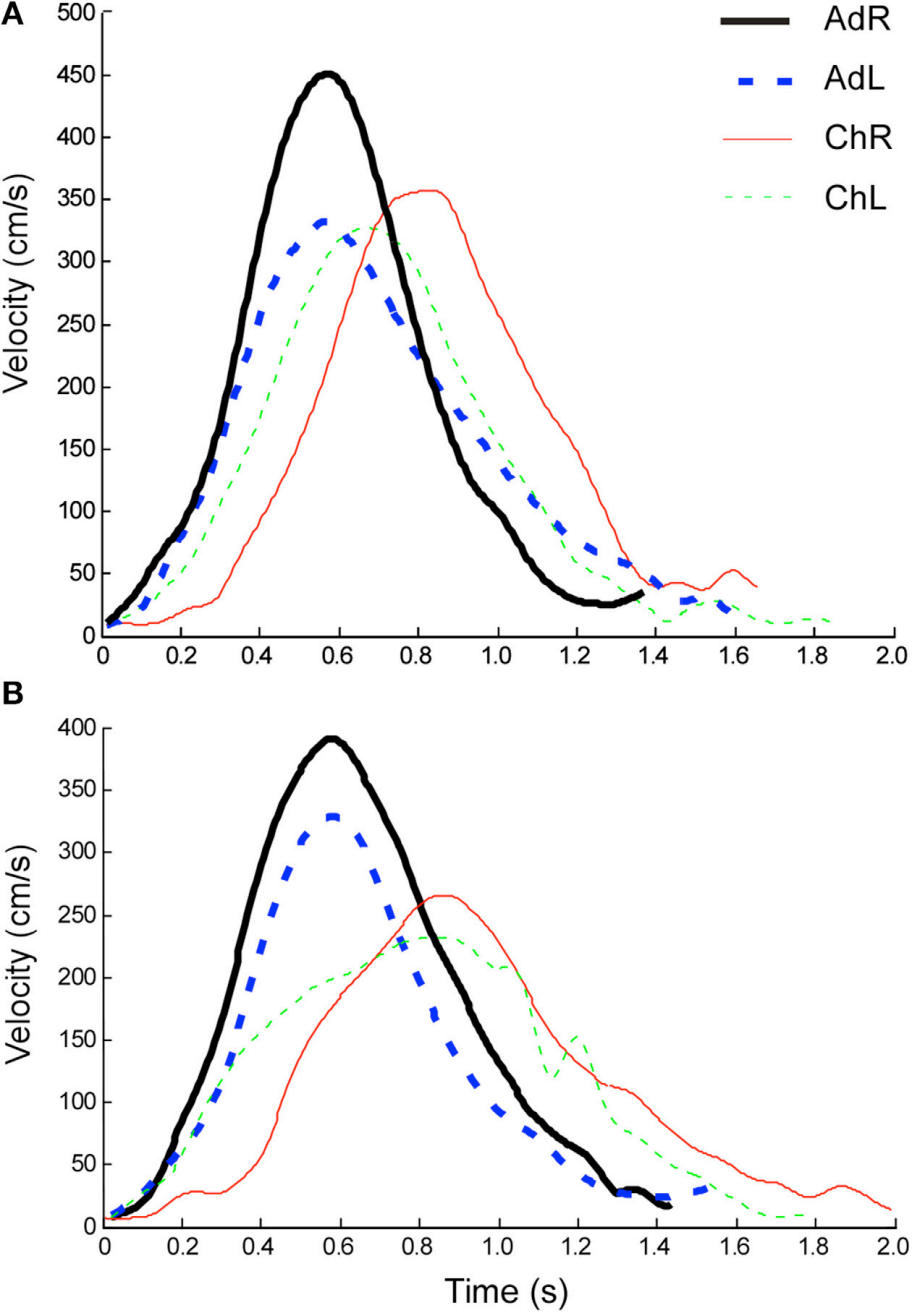
**Representative hand velocity (cm/s) curves of single trials for the reaching (A) and transporting (B) components of the task, comparing hand profiles between age groups. Ad, adults; Ch, children; R, right; L, left**.

Representative curves of hand velocity (wrist marker) of the right and left hands are shown in Figure 5, comparing profiles between adults and children for the reaching (A) and transporting (B) components. Analysis of movement kinematics was made through a Four Way linear mixed model, 2 (group: control × experimental) × 2 (age: children × adults) × 3 (test: pretest × posttest × retention) × 2 (hand: right × left), ANOVAs with repeated measures on the last two factors. Analyses of kinematic variables of the components of reaching and transporting the card were made separately. Table 1 presents descriptive kinematic data, comparing the right and left hands across tests. Results indicated absence of significant main effects or interactions associated with lateralized practice in the experimental groups in all analyses, as presented in the following.

**Table 1:** **Comparison of kinematic landmarks between the left and right hands across tests**.

	**Reaching**						**Transporting**					
	**Left**			**Right**			**Left**			**Right**		
	**Pretest**	**Posttest**	**Retention**	**Pretest**	**Posttest**	**Retention**	**Pretest**	**Posttest**	**Retention**	**Pretest**	**Posttest**	**Retention**
**EXPERIMENTAL ADULTS**												
Movement time (s)	0.93 (0.16)	0.95 (0.12)	0.92 (0.15)	0.89 (0.15)	0.92 (0.12)	0.88 (0.14)	1.34 (0.27)	1.40 (0.22)	1.21 (0.23)	1.09 (0.17)	1.10 (0.18)	1.06 (0.23)
Straightness score	0.71 (0.08)	0.73 (0.06)	0.68 (0.10)	0.66 (0.10)	0.71 (0.80)	0.68 (0.08)	0.51 (0.15)	0.50 (0.16)	0.51 (0.21)	0.59 (0.18)	0.58 (0.19)	0.63 (0.14)
Movement units (*n*)	1.08 (0.13)	1.03 (0.08)	1.03 (0.08)	1.11 (0.22)	1.03 (0.08)	1.03 (0.08)	2.04 (0.78)	1.77 (0.47)	1.48 (0.31)	1.39 (0.64)	1.35 (0.51)	1.22 (0.34)
**CONTROL ADULTS**												
Movement time (s)	1.00 (0.13)	1.04 (0.18)	0.98 (0.15)	1.04 (0.22)	0.94 (0.18)	0.96 (0.20)	1.41 (0.21)	1.55 (0.34)	1.39 (0.39)	1.16 (0.29)	1.14 (0.29)	1.24 (0.19)
Straightness score	0.65 (0.11)	0.69 (0.10)	0.65 (0.08)	0.73 (0.10)	0.69 (0.10)	0.67 (0.08)	0.57 (0.15)	0.58 (0.15)	0.57 (0.13)	0.60 (0.19)	0.62 (0.18)	0.63 (0.16)
Movement units (*n*)	1.15 (0.14)	1.13 (0.33)	1.06 (0.11)	1.29 (0.38)	1.06 (0.11)	1.00 (0.00)	1.97 (0.85)	2.13 (1.06)	1.61 (0.44)	1.25 (0.33)	1.55 (0.47)	1.53 (0.52)
**EXPERIMENTAL CHILDREN**												
Movement time (s)	1.12 (0.23)	1.12 (0.21)	1.14 (0.26)	1.06 (0.19)	1.06 (0.24)	0.97 (0.30)	1.71 (0.36)	1.80 (0.45)	1.91 (0.46)	1.39 (0.33)	1.38 (0.29)	1.49 (0.36)
Straightness score	0.64 (0.07)	0.67 (0.07)	0.68 (0.12)	0.68 (0.07)	0.67 (0.10)	0.59 (0.05)	0.44 (0.18)	0.44 (0.15)	0.52 (0.19)	0.54 (0.17)	0.58 (0.14)	0.53 (0.17)
Movement units (*n*)	1.83 (0.75)	1.96 (0.79)	2.89 (0.74)	1.69 (1.19)	1.61 (0.58)	1.60 (0.63)	3.58 (1.49)	3.42 (1.57)	3.58 (1.32)	2.34 (0.96)	2.60 (0.96)	2.07 (0.63)
**CONTROL CHILDREN**												
Movement time (s)	1.15 (0.27)	1.31 (0.44)	1.10 (0.21)	1.04 (0.20)	1.04 (0.20)	1.11 (0.24)	1.73 (0.41)	1.59 (0.45)	1.84 (0.43)	1.43 (0.22)	1.60 (0.39)	1.42 (0.28)
Straightness score	0.64 (0.10)	0.67 (0.15)	0.70 (0.05)	0.65 (0.08)	0.65 (0.06)	0.62 (0.10)	0.61 (0.09)	0.55 (0.13)	0.52 (0.13)	0.67 (0.11)	0.56 (0.16)	0.59 (0.12)
Movement units (*n*)	1.68 (0.77)	1.70 (0.59)	1.62 (0.71)	1.30 (0.23)	1.31 (0.57)	1.45 (0.71)	3.22 (1.43)	2.54 (1.21)	4.07 (2.68)	2.29 (1.07)	2.62 (0.95)	2.36 (0.82)

*Average values with standard deviation shown in parenthesis, separately for the reaching and transporting components*.

### Reaching for the card

Analysis of movement time revealed significant main effects of group [*F*_(1, 34)_ = 4.38, *p* = 0.04], due to longer movement times in the control (*M* = 1.06 s, *SD* = 0.24) than in the experimental (*M* = 0.99 s, *SD* = 0.21) groups; age [*F*_(1, 34)_ = 25.47, *p* = 0.001], indicating that children (*M* = 1.10 s, *SD* = 0.25) had longer movement times than adults (*M* = 0.95 s, *SD* = 0.16); and hand [*F*_(1, 34)_ = 5.59, *p* = 0.02], due to longer movement times of movements performed with the left (*M* = 1.06 s, *SD* = 0.24) than the right (*M* = 0.99 s, *SD* = 0.20) hand. Analysis of straightness revealed a significant main effect of age [*F*_(1, 34)_ = 5.42, *p* = 0.02], due to the fact that children (*M* = 0.65, *SD* = 0.09) presented lower values than adults (*M* = 0.68, *SD* = 0.09). Analysis of number of movement units revealed a significant main effect of age [*F*_(1, 34)_ = 97.20, *p* = 0.001], indicating that children (*M* = 1.88, *SD* = 0.25) presented an increased number of movement units than adults (*M* = 1.04, *SD* = 0.84).

### Transporting the card

Analysis of movement time revealed significant main effects of age [*F*_(1, 34)_ = 63.44, *p* = 0.001], indicating that children (*M* = 1.61 s, *SD* = 0.40) presented longer movement times than adults (*M* = 1.26 s, *SD* = 0.29); and hand [*F*_(1, 34)_ = 41.00, *p* = 0.001], due larger values for the left (*M* = 1.57 s, *SD* = 0.41) than for the right (*M* = 1.29 s, *SD* = 0.31) hand. Analysis of straightness revealed significant main effects of group [*F*_(1, 34)_ = 7.38, *p* = 0.007], indicating that controls (*M* = 0.59, *SD* = 0.14) presented higher values than the experimental (*M* = 0.53, *SD* = 0.17) groups; and hand [*F*_(1, 34)_ = 9.74, *p* = 0.002], due to higher values for movements performed with the right (*M* = 0.59, *SD* = 0.16) than with the left (*M* = 0.53, *SD* = 0.15) hand. Analysis of number of movement units revealed significant main effects of age [*F*_(1, 34)_ = 79.10, *p* = 0.001], indicating that children (*M* = 2.84, *SD* = 1.47) presented an increased number of movement units than adults (*M* = 1.50, *SD* = 0.74); and hand [*F*_(1, 34)_ = 27.51, *p* = 0.001], due to increased values for movements performed with the left (*M* = 2.56, *SD* = 1.55) than with the right (*M* = 1.77, *SD* = 0.94) hand.

## Discussion

The present experiment was designed to evaluate the effect of use of the non-preferred left hand in the practice of several tasks on manual preference to perform motor tasks different from those practiced. Evaluation was made in age groups acknowledged to have consistent manual preference, comparing tasks and spatial arrangements inducing distinct frequencies of right/left hand use. At a descriptive level, analysis showed an overall trend toward increased preference of the left hand following practice, with some cases of shift to global left hand preference in both age groups. Statistical analysis indicated that the effect of left hand practice on manual preference reached significance at specific tasks/positions. For the adults, increased preference for the left hand was observed at the midline position for the simple task, while for the children use of the left hand during practice modulated manual preference in the two rightmost target positions in the complex task. The observed effects were persistent over 20 days of rest, and were not associated with variation of intermanual performance asymmetry. Results are in agreement with the expected diffusion of manual preference acquired through systematic hand use over manual preference of distinct motor tasks.

A preliminary point to consider in the results is that power of modulation of manual preference by left hand practice was less evident than has been found in previous investigation in children (Teixeira et al., 2010) and adults (Teixeira and Okazaki, 2007), with significant effects only at specific probing conditions for each age group. Limited modulation of manual preference in the present results might be thought to derive from different points. First, even though we used reaching and manipulative tasks during practice, just a few of those tasks were strictly similar to the tasks employed to probe manual preference. As we showed in previous results, diffusion of manual preference between tasks was stronger when the probing task was similar in its sequential structure to that practiced (Teixeira and Okazaki, 2007). For another probing task requiring a different sequential structure the diffusion of manual preference was less evident. From these results, it seems that similarity between practice and probing tasks is a factor limiting the diffusion of manual preference resulting from lateralized practice. Second, we tested age groups expected to be consistent in the use of the right hand, a feature particularly evident in the children at the age employed in this experiment (cf. Bryden and Roy, 2006; Doyen et al., 2008; Hill and Khanem, 2009). This aspect may have attenuated a more generalized effect of left hand practice on manual preference as has been previously found in young children (Teixeira et al., 2010). It is plausible that, as young children are inconsistent in manual preference (Gesell and Ames, 1947; Carlier et al., 2006; Leconte and Fagard, 2006; Doyen et al., 2008; Hill and Khanem, 2009; Bryden et al., 2011), they are more strongly affected by using a single hand. By considering the lack of task specificity and that we tested groups of consistent manual preference, on the other hand, results of persistent modulation of manual preference by systematic use of the left hand indicates the power of lateralized experiences in the development of a generalizing bias in hand selection for motor performance. Some noticeable cases were those in which manual preference in the probing tasks was shifted toward the left hand, as indicated by the global score across card positions, in both age groups. This result suggests that consistent use of the left hand to perform motor tasks can induce not only a more frequent use of that hand but also its prevalent use over the globally preferred right hand. Further on this point, we highlight the finding that such a persistent modulation of manual preference over several days of rest was achieved from a moderate amount of practice regarding the number of motor experiences accumulated with the preferred right hand in daily living activities. These findings support the notion that manual preference is a dynamic component of motor behavior continuously open to change.

Left hand practice induced modulation of manual preference differently between children and adults. For the adults the most noticeable change of manual preference following practice took place at the midline. Although the effect of practice was significant for the simple task only, the same trend was observed also for the complex task. This result is consistent with a previous finding showing that increased use of the non-preferred hand as a result of feedback manipulation in adults is more evident at the central in comparison with lateral positions (Stoloff et al., 2011). We interpret this result in the light of previous findings suggesting that there is a competition between motor plans to perform an action with either the right or the left hand (Oliveira et al., 2010). In situations in which the target is located at a lateral position in the workspace, proximity between the hand and the target (Gabbard and Helbig, 2004; Helbig and Gabbard, 2004) and biomechanical constraints (Carey et al., 1996; Bryden and Huszczynski, 2011; Kim et al., 2011) introduce a contextual transient bias in hand selection. At the midline position, however, there is no physical advantage for either hand. Then, at this position higher frequency of use of a single hand can be thought to express more clearly a relatively permanent global bias of hand selection. From the comparison between age groups, it becomes apparent that adults attribute a larger weight to contextual biomechanical constraints than to the global bias in hand selection as compared to children. This conclusion is consistent with the finding that adults privilege a comfort state in hand selection in detriment of the global lateral bias (cf. Coelho et al., 2014). For the children, increased manual left hand preference at the two rightmost target positions after practice is consistent with previous findings in younger children showing a more evident modulation of manual preference due to left hand practice in targets positioned in the right hemispace (Teixeira et al., 2010). However, even though increased preference of the left hand after practice was found to be significant at the two rightmost positions only, a similar trend can be observed for the other right sided positions. This result seems to reflect a particular characteristic of modulation of manual preference by use in the children. For target positions in the right hemispace there are two physical factors biasing selection of the right hand, namely target proximity (Gabbard and Helbig, 2004; Helbig and Gabbard, 2004) and mechanical efficiency (Carey et al., 1996; Bryden and Huszczynski, 2011; Kim et al., 2011). Combination of these two contextual factors with a global bias to select the preferred right hand seems to be responsible for an almost exclusive use of that hand to reach for and manipulate right-sided targets before practice, which is in consonance with previous findings (cf. Bryden and Roy, 2006; Carlier et al., 2006; Doyen et al., 2008; Hill and Khanem, 2009). The fact that the children increased frequency of use of the left hand in right-sided positions following practice suggests that the global lateral bias toward using the left hand was strong enough to overcome the contextual spatial-related bias of those target positions inducing selection of the right hand. The finding that use-dependent modulation of manual preference took place in the complex task in the children suggests that they became highly confident in using their left hand even when the task required increased manipulation accuracy. This finding is contradictory with the supposition that the non-preferred hand is more probably used in tasks requiring simple movements. It becomes apparent that following practice the children became confident in using their left hand to perform tasks requiring crossing the midline and demanding increased accuracy. This characteristic sharply contrasts with the pretest results, in which not a single case was observed of reaching for the rightmost card positions with the left hand. Hence, an important point emerging from our results is that children at this age, although reported to be highly consistent in the selection of their right hand (e.g., Bryden and Roy, 2006), were shown to be malleable to the effect of hand use. From this finding, it might be thought that some environmental factor taking place regularly in daily living experiences at this age, like increased unimanual use for handwriting, leads to high consistency in the preference of the right hand to perform several other motor tasks.

A point to be underscored in the results was that increased use of the left hand following practice was not paralleled by a change of intermanual performance asymmetry in movement kinematics. Absence of change in the between-hand relationship of motor performance following left hand practice was foreseen at the experiment outset, since the practiced motor tasks were distinct from those used to evaluate manual preference and there was no emphasis on improvement of motor performance during practice. Modulation of manual preference by means of left hand practice, then, was shown to vary as a consequence of lateralized use rather than to development of manual asymmetry favoring the left hand. From this result, it is implausible that modulation of manual preference have been a consequence of an improved capacity to perform the probing tasks with the left hand, or due to less attentional effort due to movement automatization. This is an important point for a theory of lateralization of motor behavior, since prevalent models of human laterality are based on the assumption that manual preference derives from cerebral hemisphere dominance and associated intermanual performance asymmetry (Annett, 1972; Levy and Nagylaki, 1972; McManus, 1985). Przybyla et al. (2013) have presented evidence suggesting that relative better performance of the non-dominant left hand in aiming movements performed in the absence of visual feedback biases manual preference for that hand. However, it should be considered the possibility that higher frequency of use of the left hand in Przybyla's results may have been due not to performance improvement *per se*, but to the sense of higher likelihood of success when using the non-dominant left hand. This interpretation is based on Stoloff et al. (2011) findings that perception of greater proficiency of the non-preferred hand, without effective improvement of movement control, leads to higher probability of its use to perform a motor task. Stoloff's results suggest that hand selection in a given trial is biased by confidence of success when using a given hand, established from the history of previous experiences. In line with this rationale, we interpret our results from the perspective that use of the non-preferred left hand in different manual tasks increased the confidence on that hand to perform movements requiring accuracy. The finding that the practiced tasks were not specific for the evaluation of manual preference suggests that increased confidence on one hand diffuses over distinct motor tasks, inducing a global bias in hand selection. We propose that increased confidence on the capacity to control proficiently movements of a given hand leads to a predisposition to plan movements for that hand, even in cases that a task is performed for the very first time. We conceptualize confidence on hand performance as a high-level component of movement organization affecting decision making about hand selection in a variety of motor tasks, which we name as “diffusion.” Convergent to this proposition, Sabaté et al. (2004) have shown that in tasks requiring rapid finger movements, different movement times between hands in physical execution are expressed also in movement imagery. Sabaté has proposed that the brain scans the motor competence of the limbs, adjusting the planning of future movements to their estimated capability. This level of movement organization, then, is able to have a pervasive effect on manual preference. From this notion, it is possible that the expected poor performance with the non-preferred hand in daily living situations leads to planning movements for the preferred hand, leading to a higher frequency of its use.

As concluding remarks, we underscore the finding that modulation of manual preference following left hand practice was shown to be due to left hand use *per se* rather than to improvement of proficiency in the performance with the left hand. This finding indicates that manual preference is not fundamentally associated with intermanual performance asymmetry. However, as manual skill rapidly improves at the beginning of lateralized practice, consistent selection of a single hand to perform a task is expected to lead to intermanual performance asymmetry favoring either the right or the left hand (cf. Teixeira, 1999, 2000), reinforcing the confidence on the selected hand for motor performance. The finding that modulation of manual preference was achieved through practice of non-specific motor tasks supports the conceptualization of diffusion of manual preference from the practiced over different movements. Although children and adults were affected in particular ways by left hand practice, both age groups showed malleability to modulate manual preference as a result of the recent history of differential use between the hands. As these age groups are acknowledged to have consistent manual preference for the right hand, we consider to have made a strict test of the effect of hand use on manual preference in this experiment. Our results, then, offer an alternative interpretation for lateralization of behavior, which may be based on systematic single hand use in a set motor tasks and diffusion of the resultant manual preference over several distinct motor actions.

### Conflict of interest statement

The authors declare that the research was conducted in the absence of any commercial or financial relationships that could be construed as a potential conflict of interest.

## Footnotes

^1^The term “complexity” is used here to denote use of attentional resources due to accuracy demand of the task.

^2^ We draw the reader's attention to our use of the term “practice” to mean a series of systematic motor activities to provide practical experiences without the purpose of motor learning, as this term usually implies.
